# The impact of non-pharmaceutical interventions on community non-SARS-CoV-2 respiratory infections in preschool children

**DOI:** 10.1186/s12887-024-04686-2

**Published:** 2024-04-01

**Authors:** Bianca Klee, Sophie Diexer, Johannes Horn, Susan Langer, Marie Wende, Diego Ortiz, Agata Bielecka, Till Strowig, Rafael Mikolajczyk, Cornelia Gottschick

**Affiliations:** 1 Institute for Medical Epidemiology, Biometrics and Informatics, Interdisciplinary Centre for Health Sciences, Medical Faculty of the Martin Luther University Halle-Wittenberg, Halle (Saale), Germany; 2 Helmholtz Centre for Infection Research, Braunschweig, Germany; 3 German Centre for Infection Research (DZIF), Partner Site Hannover-Braunschweig, Braunschweig, Germany; 4 Hannover Medical School, Cluster of Excellence RESIST (EXC 2155), Hannover, Germany

**Keywords:** Respiratory tract infections, Birth cohort study, Non-pharmaceutical interventions

## Abstract

**Background:**

Effects of non-pharmaceutical interventions during the pandemic were mainly studied for severe outcomes. Among children, most of the burden of respiratory infections is related to infections which are not medically attended. The perspective on infections in the community setting is necessary to understand the effects of the pandemic on non-pharmaceutical interventions.

**Methods:**

In the unique prospective LoewenKIDS cohort study, we compared the true monthly incidence of self-reported acute respiratory infections (ARI) in about 350 participants (aged 3–4 years old) between October 2019 to March 2020 (pre-pandemic period) and October 2020 to March 2021 (pandemic period). Parents reported children’s symptoms using a diary. Parents were asked to take a nasal swab of their child during all respiratory symptoms. We analysed 718 swabs using Multiplex PCR for 25 common respiratory viruses and bacteria.

**Results:**

During the pre-pandemic period, on average 44.6% (95% CI: 39.5–49.8%) of children acquired at least one ARI per month compared to 19.9% (95% CI: 11.1–28.7%) during the pandemic period (Incidence Rate Ratio = 0.47; 95% CI: 0.41–0.54). The detection of influenza virus decreased absolute by 96%, respiratory syncytial virus by 65%, metapneumovirus by 95%, parainfluenza virus by 100%, human enterovirus by 96% and human bocavirus by 70% when comparing the pre-pandemic to the pandemic period. However, rhinoviruses were nearly unaffected by NPI. Co-detection (detection of more than one virus in a single symptomatic swab) was common in the pre-pandemic period (222 of 390 samples with viral detection; 56.9%) and substantially less common during the pandemic period (46 of 216 samples; 21.3%).

**Conclusion:**

Non-pharmaceutical interventions strongly reduced the incidence of all respiratory infections in preschool children but did not affect rhinovirus.

## Background

The World Health Organisation declared a global pandemic on March 11, 2020, which was caused by the severe acute respiratory syndrome coronavirus type 2 (SARS-CoV-2) and the resulting coronavirus disease of 2019 (COVID-19) [1]. SARS-CoV-2 appeared to affect children less often during the early stages of the COVID-19 pandemic due to mostly asymptomatic or mildly symptomatic cases [2, 3]. However, subsequent studies showed that children are as likely as adults to acquire and transmit SARS-CoV-2 [4–6]. During COVID-19–related lockdown periods, the German government issued, among other non-pharmaceutical interventions (NPI) (i.e. physical distancing, stay-at-home orders, lockdown of non-essential shops, mandatory mask wearing), the closure of schools and day care centres. Given that all respiratory viruses are transmitted via similar mechanisms – aerosols or droplets – NPI targeting SARS-CoV-2 also had the potential to reduce infections by respiratory viruses other than SARS-CoV-2 [7–10]. In Germany, for example, flu season suddenly stopped in week 14 during winter 2019/20 (after the first COVID-19 lockdown was issued in week 12) [11], and an almost undetectable flu season followed during winter 2020/21 (in a period of stepwise increasing restrictions) [12]. In children, studies using data based on hospital records or registers from Australia, Austria, Finland and Japan show that the incidence decreased for different respiratory viruses, e.g. influenza, respiratory syncytial virus and metapneumovirus but not for rhino-/enterovirus and adenovirus during lockdown periods [8, 13–15].

So far, studies have shown reduced frequencies of acute respiratory infections (ARI) and changed viral and bacterial presence during COVID-19 lockdown periods using mostly hospital data or data based on registers from laboratories only. However, most ARI in children are not medically attended [16], and hospital based studies are related to severe infections, thus most of the ARI among preschool children are not included in previous research. In addition, the viral and bacterial profile might be disrupted in hospital-based studies. Furthermore, during the lockdown period, parents were reported to avoid seeing medical doctors for consultations, possibly further changing the viral spectrum detected in medical settings [9, 10, 17].

This study aimed to investigate the impact of COVID-19–related NPI during the winter period 2020/21 compared to the winter period 2019/20 on the occurrence of all ARI, i.e. also those not medically attended, and the respiratory viral and bacterial presence among 3- and 4-year-old children in a population-based cohort in Germany.

## Methods

### Study sample

We used data and nasal swabs from the birth cohort study LoewenKIDS (Clinicaltrials.Gov Identifier: NCT02654210) which is described in detail elsewhere [18]. Briefly, between 2014 and 2018, we enrolled 782 newborns in five German study centres (336 children in Braunschweig, 174 children in Hannover, 97 children in Bremen, 91 children in Munich and 76 children in Halle). During the first six years of their child’s life, parents are asked to fill in a daily symptom diary and take nasal swabs at each event of respiratory symptoms. The Ethics Committees of the Martin Luther-University Halle-Wittenberg (No. 2016-04), the Medical School Hannover (No. 6794) and the Ludwig Maximilian University Munich (No. 445 − 15), Germany approved the study. Parents received detailed information on the objectives of the cohort study and provided written informed consent.

### Symptom diary and ARI episode definition

Parents were asked to record symptoms and to rate their severity on a daily basis in a symptom diary. Symptoms included fever, wheezing, cough with sputum (category A) and runny nose or nasal congestion, sore throat, cough, chills, loss of appetite, increased need to sleep, increased attachment (category B) and were classified as previously described [19]. Participants aged three or four years in the times of interest with at least 80% completeness of daily entries for the symptoms were included in the analysis of the ARI episodes.

### Nasal swabs

Parents collected nasal swabs at the beginning of an ARI, which was defined as one or more symptoms of category A or two or more symptoms of category B. Swabs were put in tubes with transport media (eNat or Amies Medium) and transported via regular mail to the laboratory within 48h of collection. Specimens were stored at -80°C until further analysis. We analysed all symptomatic nasal swabs (*n* = 457) of 206 children aged 3–4 years in the period October 2019 to March 2020 (pre-pandemic period) and all swabs (*n* = 261) of 162 children aged 3–4 years in the period October 2020 to March 2021 (pandemic period). Participants did not receive the results of the nasal swab testing. There were no restrictions for the selection of nasal swabs regarding the completeness of the symptom diary.

### RNA extraction and qPCR analysis

Viral RNA was extracted from 200 μl of nasopharyngeal aspirate specimens using the Quick-DNA/RNATM Viral MagBeadkit (Zymo Research), according to the manufacturer’s instructions. We screened the samples by multiplex PCR with the AllplexTM Respiratory Panel 2–4 and AllplexTM SARS-CoV-2/FluA/FluB/RSV Assay (Seegene Germany GmbH) using the CFX96 Dx System (Bio-Rad). The samples were analysed using the CFX Manager™ Dx Software v3.1 and Seegene Viewer. All assays were carried out as described by the manufacturer’s instructions. The four panels included assays for the following respiratory viruses and bacteria: Influenza A virus (FluA), Influenza B virus (FluB), Respiratory Syncytial Virus (RSV A & B), Adenovirus (AdV), Enterovirus (HEV), Metapneumovirus (MPV), Parainfluenza 1–4 (PIV1-4), Bocavirus (HBoV), Coronaviruses (229E, OC43, NL63), Rhinovirus (HRV), SARS-CoV-2 (N gene, RdRP gene), *Bordetella parapertussis* (BPP), *Bordetella pertussis* (BP), *Chlamydophila pneumonia*e (CP), *Haemophilus influenza*e (HI), *Legionella* pneumophila (LP), *Mycoplasma pneumonia*e (MP) and *Streptococcus pneumonia*e (SP). Invalid samples with negative internal control values (*n* = 2) were not included in the analysis.

### Statistical analysis

R software (Version 4.0.5) was used for data handling and all statistical analyses. For the classification of symptoms and episodes, we used the R-package lkstaR [20]. Descriptive characteristics were summarised using counts, percentages and means, with a 95% confidence interval (95% CI). For the incidence rate, we divided the number of ARI by the number of person-months in the respective period. We calculated the incidence rate ratio by dividing the incidence rate (number of ARI per person and month) during the pandemic period by the incidence rate of participants during the pre-pandemic period.

## Results

### Characteristics of the study population

We selected all participants aged 3–4 years in the pre-pandemic and pandemic periods resulting in 323 and 368 participants, respectively. Among those, 257 participants were included in both periods. Table 1 summarises the demographic characteristics of the participants. There is a difference of the age proportion between the pre-pandemic and pandemic period because we did not recruit the same number of children each year. We received 457 nasal swabs during the pre-pandemic period and 261 nasal swabs during the pandemic period.

**Table 1:** Socio-demographics of participants enrolled in the study

Characteristics	Pre-pandemic period *N* (%)	Pandemic period *N* (%)
Number	323	368
Sex		
Female	164 (50.8)	176 (47.8)
Male	157 (48.6)	188 (51.1)
Missing	2 (0.6)	4 (1.1)
Age (in years)		
3	217 (67.2)	147 (39.9)
4	106 (32.8)	221 (60.1)
Study Region		
Braunschweig	147 (45.5)	144 (39.1)
Hannover	73 (22.6)	77 (20.9)
Halle (Saale)	17 (5.3)	45 (12.2)
Munich	40 (12.4)	48 (13.0)
Bremen	45 (13.9)	51 (13.9)
Other	1 (0.3)	3 (0.8)
Number of persons in household		
2	5 (1.5)	2 (0.5)
3	129 (39.9)	152 (41.3)
4	138 (42.7)	159 (43.2)
5	41 (12.7)	43 (11.7)
≥ 6	6 (1.9)	7 (1.9)
Missing	4 (1.2)	5 (1.4)
Socio economic status*		
Low	2 (0.6)	2 (0.5)
Middle	23 (7.1)	30 (8.2)
High	293 (90.7)	330 (89.7)
Missing	5 (1.5)	6 (1.6)
Total of submitted symptomatic nasal swabs	457	261

*According to Brandenburg social status index [64]

### Incidence and characteristics of ARI during the pre-pandemic and pandemic periods

**Fig. 1:**
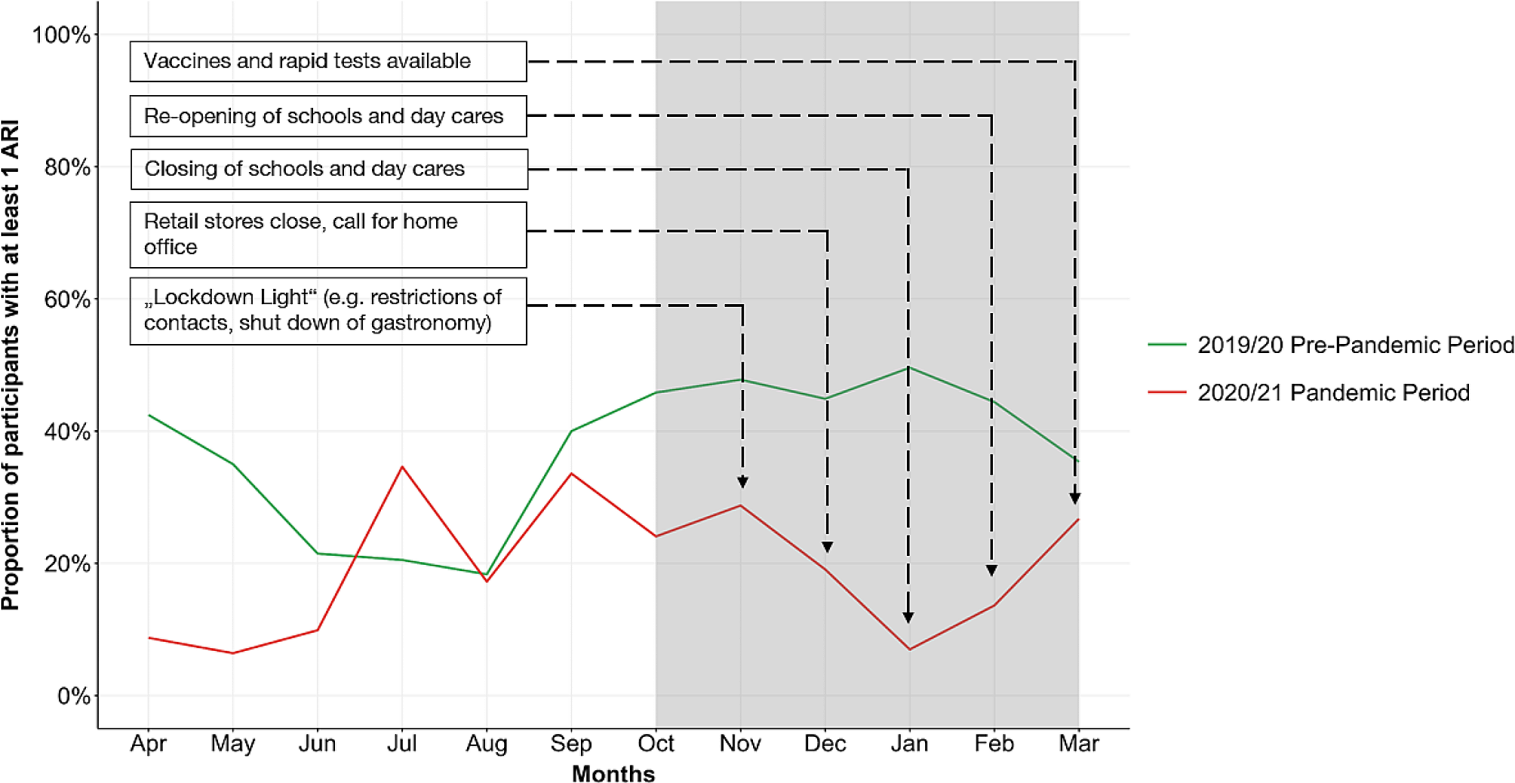
Changes in incidence of ARI during the pre-pandemic and pandemic periods. The grey box indicates the winter periods, which were selected for further analysis

We detected 616 ARI among 222 children during the pre-pandemic winter and 259 ARI among 144 children during the pandemic winter. In the pre-pandemic period, on average 44.6% (95% CI: 39.5–49.8%) of the participants had at least one ARI per month, whereas this number decreased to 19.9% (95% CI: 11.1–28.7%) during the pandemic period. The incidence rate of ARI among children in the pre-pandemic period was 2.8 ARI per person in six months and therefore 0.47 (95% CI: 0.41–0.54) times as high as the rate among children during the pandemic period (1.3 ARI per person in six months). This decrease in the proportion of participants with at least one ARI paralleled the start of NPI in autumn 2020 and the increasing intensity of the interventions (Fig. 1). The lowest incidence, with 6.9% of participants with at least one ARI per month, occurred in January 2021, which paralleled the strictest COVID-19 measures. After the easing of restrictions in February 2021, we observed an increase of ARI. In March 2021, the proportion of ARI reached almost the level observed before the pandemic.

During the pre-pandemic winter, an ARI episode lasted on average 12.3 days (95% CI: 11.3–13.3 days) compared to 8.6 days (95% CI: 7.5–9.7 days) during the pandemic winter (Table 2). Symptom patterns were similar for both periods. During the pre-pandemic period, 14.0% (95% CI: 10.8–17.2%) of all participants reported ARI were medically attended and 10.0% (95% CI: 3.5-16.5%) during the pandemic period.

**Table 2:** Length of ARI and presence of symptoms during pre-pandemic and pandemic periods

	Pre-pandemic period	Pandemic period
Length of ARI in days (Mean, 95% CI)	12.3 (11.3–13.4)	8.6 (7.5–9.7)
Proportion of days of ARI with …(%, 95% CI)		
runny nose	71.2 (70.4–71.9)	70.8 (69.7–71.6)
cough	61.9 (59.7–63.8)	56.5 (53.2–59.1)
increased attachment	14.3 (13.7–14.9)	17.3 (16.6–20.2)
increased need to sleep	13.5 (12.8–14.1)	11.3 (9.5–12.6)
loss of appetite	12.2 (11.4–12.8)	9.7 (8.2–10.9)
fever	7.5 (6.9–7.9)	5.4 (4.6–6.1)
wheezing	3.3 (2.3–4.2)	4.1 (1.4–6.1)
sore throat	3.1 (2.5–3.6)	4.6 (3.3–5.6)
chills	0.8 (0.5–1.1)	1.0 (0.4–1.5)
Proportion of all ARI that were medically attended (%, 95% CI)	14.0 (10.8–17.2)	10.0 (3.5–16.5)
Proportion of ARI with hospital admissions	0.6%	0.0%

### Distribution of viruses in symptomatic nasal swabs in the pre-pandemic and pandemic periods

**Fig. 2:**
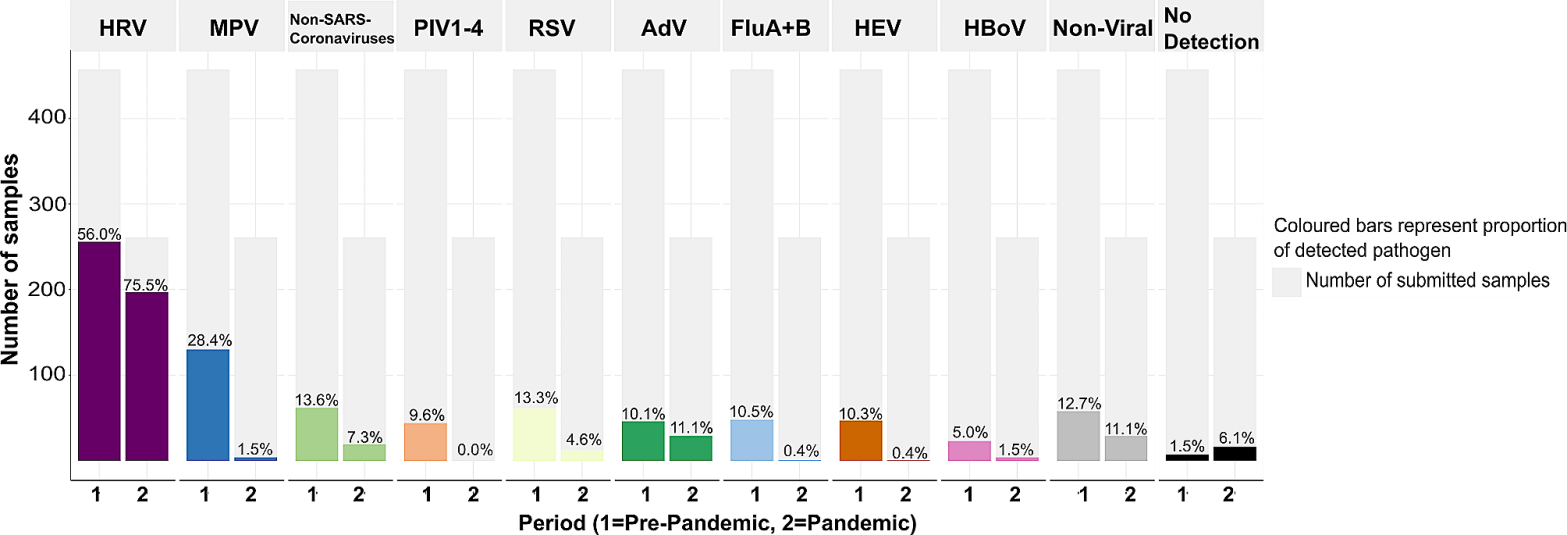
Prevalence (%) of viruses and bacteria detected in both periods in relation to the number of submitted samples. (HRV: Human Rhinovirus; MPV: Metapneumovirus; Non-SARS-Coronaviruses: Human Coronaviruses 229E, OC43, NL63; PIV1-4: Parainfluenza Viruses 1–4; RSV: Respiratory Syncytial Virus; AdV: Adenovirus; FluA + B: Influenza A virus and Influenza B virus; HEV: Human Enterovirus; HBoV: Human Bocavirus; Non-viral includes all bacterial strains: Bordetella parapertussis, Bordetella pertussis, Chlamydophila pneumoniae, Haemophilus influenzae, Legionella pneumophila, Mycoplasma pneumoniae and Streptococcus pneumonia)

We analysed 455 nasal swabs from 206 children for the pre-pandemic period and 261 nasal swabs from 162 children for the pandemic period using multiplex PCR. We did not detect any bacteria or viruses in 23 samples (3.2%). While the absolute incidence for all tested viruses decreased, the fraction of adenoviruses and bacteria (non-viral) among all swabs remained at the same level, and the fraction of samples containing rhinoviruses even increased (Fig. 2). Other viruses (MPV, PIV1-4, FluA + B, HEV) were not detected during the pandemic period. We did not detect SARS-CoV-2 in any sample.

Changes in the distribution of viruses and bacteria were accompanied by a substantially decreased number of co-detections. Overall, we detected only viral strains in 57 samples (8.2%), only bacterial strains in 87 samples (12.6%) and viral plus bacterial strains in 549 samples (79.2%, Table 3). During the pre-pandemic period, we observed that 56.9% of all samples had a viral co-detection (more than one virus identified per sample); this fraction decreased to only 21.3% during the pandemic period. While we found up to six viruses in one sample during the pre-pandemic period, we only found up to three viruses in one sample during the pandemic period. The most common combinations for simultaneous presence of viruses were HRV together with MPV (21.9%) or together with AdV (20.8%). For the samples with only bacterial presence, more than 50% of samples had a double detection with HI and SP in both periods. Co-detection of viruses and bacteria were more common in the pre-pandemic period (81.9%) compared to the pandemic period (67.4%). Among them, most samples with viruses showed a co-detection with the bacteria HI and SP, followed by a co-detection with either HI or SP.

**Table 3:** Number and characteristics of viral and bacterial co-detection for analysed samples in pre-pandemic and pandemic periods (BPP: *Bordetella parapertussis*; BP: *Bordetella pertussis;* CP: *Chlamydophila pneumonia*; HI: *Haemophilus influenza*; LP: *Legionella* pneumophila; MP: *Mycoplasma pneumonia*; SP: *Streptococcus pneumonia*)

	Pre-pandemic Period	Pandemic period
**Samples with ≥ 1 detected virus**		
Total number / all valid samples (%)	390/455 (85.7)	216/261 (82.8)
Average number of detected viruses per sample (95% CI)	1.8 (1.7–1.9)	1.2 (1.1–1.3)
Number of detected viruses per sample (%)		
1	168 (43.1)	170 (78.7)
2	137 (35.1)	41 (19.0)
3	66 (16.9)	5 (2.3)
4	15 (3.8)	0 (0.0)
5	3 (0.8)	0 (0.0)
6	1 (0.3)	0 (0.0)
**Samples with only bacterial presence**		
Total number / all valid samples (%)	58/455 (12.7)	29/261 (11.1)
Number of samples with SP	21 (36.2)	8 (27.6)
Number of samples with HI	5 (8.6)	6 (20.7)
Number of samples with SP + HI	30 (51.7)	15 (51.7)
Number of samples with BPP	2 (3.4)	0 (0.0)
**Samples with only viral presence**		
Total number / all valid samples (%)	17/455 (3.7)	40/261 (15.3)
**Samples with viral and bacterial presence**		
Total number / all valid samples (%)	373/455 (81.9)	176/261 (67.4)
Virus + HI	68 (18.2)	30 (17.0)
Virus + SP	25 (6.7)	38 (21.6)
Virus + SP + HI	270 (72.4)	107 (60.8)
Virus + BPP	2 (0.5)	0 (0.0)
Virus + BPP/MP + HI	2 (0.5)	0 (0.0)
Virus + BPP/MP + HI + SP	6 (1.6)	1 (0.6)

## Discussion

Our results show that the COVID-19 pandemic-related NPI strongly reduced the number of self-reported ARI in healthy 3- and 4-year-old infants in Germany. NPI suppressed the spread of most seasonal viruses except HRV.

The observed reduction of ARI incidence during the NPI is in line with other studies focusing on the paediatric population in hospitals or using register data [8, 21, 22]. The reduction is likely to be caused by various NPI, although the impact of individual components is unclear. Respiratory infections, especially in early childhood, are very common and cause not just high socioeconomic burdens [23] but are also associated with the development of chronic diseases later in life [24]. However, fewer infections during NPI likely mean a reduced immunity afterwards. In some parts of the world, a strong atypical resurgence of respiratory infections, like RSV, was observed during summer months for children after easing NPI [25–27]. The high proportion of susceptible individuals and the lack of exposure to certain viruses and bacteria could affect timing and severity of future ARI seasons [28].

We found that the prevalence of viral strains changed before and during NPI with suppression of severe viruses such as FluA + B, PIV1-4, RSV and MPV. The natural season period of some viruses was interrupted through the NPI. For example MPV, which is normally detected in late winter and spring [29], was no longer detectable during the pandemic period [30, 31]. However, some studies reported an off-season outbreak of MPV from May to July 2021 [32, 33]. Similarly, RSV, which mostly occurs between December to March each year [29], could rarely be found during the pandemic period [30, 34–36]. Many hospitals, however, registered a massive off-season outbreak during summer months [27, 30, 34]. Interestingly, influenza virus infection incidence remained low during summer months [37, 38], this could indicate that climatic conditions and seasonality differentially affects the various viruses. In contrast, HRV was less affected by the NPI. HRV infections are highly common in children [39] and can cause light symptoms, which are usually associated with a common cold [40]. However, HRV can also cause severe symptoms leading to lower respiratory tract infections such as bronchitis [41]. In our study, HRV infections usually showed only mild symptoms.

Our findings from a population-based setting are in line with recent reports of registers or hospital data published in various countries which all describe that HRV circulation was unchanged during NPI to control the spread of SARS-CoV-2 [9, 13, 42, 43], but we also detected similar levels of AdV before and during the pandemic. This suggests that non-enveloped viruses were less affected by NPI. A major reason could be the stability of non-enveloped viruses, (e.g., HRV has been proven to be more resistant to detergents for hand washing [44] or disinfectants [45]). HRV and AdV are more effective than other airborne viruses at using the indirect transmission pathway (e.g. hand-to-hand contact followed by self-inoculation) [46–48]. This might explain the high prevalence of HRV in children, as the hygiene behaviour is not yet well established [49]. However, we detected a strong decline in HEV, which also belongs to the non-enveloped viruses. Thus, a comprehensive explanation of our findings is still lacking, and further research is needed to better understand the circulation of enveloped and non-enveloped viruses during NPI. It has been also proposed that surgical masks might not prevent the transmission of HRV [50]. On the other hand, it was discussed that HRV might display some form of colonization and thus be present independently of infection symptoms [51, 52]. If HRV were part of the microbiome, then the fraction of symptomatic swabs without a responsible virus would substantially increase, particularly during the pandemic period when fewer co-detections were present. However, we detected HRV without co-detections in the symptomatic nasal swabs especially during the pandemic period, which points towards HRV being the causative virus in our samples. There is also the possibility that we detected different HRV species. There is evidence that HRV-A and HRV-C are frequently associated with more severe infections, while HRV-B is often associated with asymptomatic infections [53, 54]. However, we did not analyse the species.

The proportion of viral co-detections before the pandemic was surprisingly high compared to most previous studies where co-detection rates up to 40% [55, 56] were reported. However, most of those studies were conducted in hospital settings with severely infected children so that virus patterns might be altered compared to the LoewenKIDS’ community setting. In healthy children, higher co-detections may be normal; however, literature about co-detection in community settings is very limited. An explanation for co-detection could be that some viral shedding is prolonged although the infection already passed. It was also shown that some viruses (e.g., HRV) are able to colonise the nasal microbiome and are therefore non-pathogenic [51, 52]. During the pandemic period, viral co-detection was markedly reduced, which reflects the decline of most viruses.

A recent systematic review and meta-analysis showed that viral co-detections in children do not have an impact on the severity of the disease, where mostly hospital-based studies with clinical outcomes were included [57]. In our analysis, we also did not observe any change in severity of symptoms.

The bacterial presence was not affected by NPI, which is supported by findings for pneumococcal carriage [58, 59]. Detection of viral and bacterial strains in the same sample was rather common, and among them, almost all samples yielded bacterial co-detections with SP and HI. Asymptomatic nasopharyngeal carriage in healthy preschool children has been documented, 64% for HI [60] and at least 50% for SP [61], meaning that children are mostly colonised with those acting commensals after entering daycare [62]. Most of the children in our cohort entered day care in the second year of life [19] potentially explaining the high prevalence of HI and SP in our samples.

### Strengths and limitations

This study has several strengths, including the utilisation of data from the prospective, longitudinal birth cohort study LoewenKIDS in a community setting [63]. Parents reported symptoms on a daily basis making it possible to evaluate the true disease burden, because mild diseases are recorded even if a physician was not consulted. Furthermore, parents provided symptomatic nasal swabs whenever the child had symptoms of ARI. Multiplex PCR including 25 major respiratory viruses and bacteria is unusual for regular care; therefore, our study provides a valuable insight into the viral prevalence of ARI during both winter seasons in a healthy paediatric cohort.

This study also has several limitations. First, we cannot exclude that the NPI and the pandemic situation in general had an impact on our participants. It might be possible that parents were more sensitive to perceive symptoms, and we overestimated the number of ARI during the pandemic period. However, participants of our study have been trained for several years to record symptoms routinely. Second, we included nasal swabs which were taken by the parents of the children and not from a health care worker. However, we provided a detailed description on how to take the swab to minimise individual sample techniques and ensure that detection rates were equal. Third, there were ARI episodes in the symptom diary without nasal swabs, and sometimes parents sent nasal swabs without corresponding entries in the symptom diary. However, the numbers were similar in both periods. ARI characteristics of the episodes with nasal swabs did not differ from the episodes without swabs (data not shown), so the missing information should not have an impact on the results. Fourth, the participants of the LoewenKIDS cohort are mostly well educated and affluent [13], (e.g., most of the parents have high academic degrees and could probably work in a home office to avoid contact to other people). This does not reflect the majority of the population in Germany, so our results could be biased by the high socio-economic status of our study population.

## Conclusion

The introduction of strict public health measures to control the COVID-19 pandemic in Germany reduced the number of self-reported ARI. Various respiratory viruses and bacteria were differentially affected and might indicate the possibility of various pathways to improve infection control among preschool children. Effects of co-detections should be further studied.

## Data Availability

The datasets used and/or analysed during the current study are available from the corresponding author upon reasonable request.

## Abbreviations

95% CI: 95% confidence interval
AdV: Adenovirus
ARI: Acute respiratory infections
BP: Bordetella pertussis
BPP: Bordetella parapertussis
COVID: 19 Coronavirus disease 2019
CP: Chlamydophila pneumoniae
FluA: Influenza A virus
FluB: Influenza B virus
HBoV: Human Bocavirus
HEV: Enterovirus
HI: Haemophilus influenzae
HRV: Human Rhinovirus
LP: Legionella pneumophila
MP: Mycoplasma pneumoniae
MPV: Metapneumovirus
NPI Non: Pharmaceutical interventions
PIV1-4: Parainfluenza virus 1–4
RSV: Respiratory Syncytial Virus
SARS: CoV-2 Severe acute respiratory syndrome coronavirus type 2
SP: Streptococcus pneumoniae
